# High throughput saliency-based quantification of grape powdery mildew at the microscopic level for disease resistance breeding

**DOI:** 10.1093/hr/uhac187

**Published:** 2022-08-25

**Authors:** Tian Qiu, Anna Underhill, Surya Sapkota, Lance Cadle-Davidson, Yu Jiang

**Affiliations:** School of Electrical and Computer Engineering, College of Engineering, Cornell University, Ithaca, NY 14850, United States of America; United States Department of Agriculture-Agricultural Research Service, Grape Genetics Research Unit, Geneva, NY 14456, United States of America; Plant Pathology and Plant-Microbe Biology Section, School of Integrative Plant Science, Cornell University, Geneva, NY 14456, United States of America; United States Department of Agriculture-Agricultural Research Service, Grape Genetics Research Unit, Geneva, NY 14456, United States of America; Plant Pathology and Plant-Microbe Biology Section, School of Integrative Plant Science, Cornell University, Geneva, NY 14456, United States of America; Horticulture Section, School of Integrative Plant Science, Cornell University, Geneva, NY 14456, United States of America

## Abstract

Imaging-based high throughput phenotyping (HTP) systems have demonstrated promising solutions to enhance genetic understanding of grapevine powdery mildew (PM) resistance and have accelerated PM-resistant cultivar breeding. The accuracy and throughput of extracting phenotypic traits from images are still the bottleneck of modern HTP systems, especially at the microscopic level. The goal of this study was to develop a saliency-based processing pipeline for the quantification of PM infection in microscopic images and comprehensively evaluate its performance for genetic analyses. An input image was segregated into subimages that were classified as infected or healthy by a pretrained CNN classifier. Saliency maps from the classification were generated post-hoc and used for the quantification of PM infection in the input image at the pixel level without the use of mask annotations. A total of seven phenotypic traits were extracted from images collected for a biparental population. Experimental results showed that optimal combinations of convolutional neural network and saliency methods achieved strong measurement correlations (r = 0.74 to 0.75) with human assessments at the image patch level, and the traits calculated by the saliency-based processing pipeline were highly correlated (r = 0.87 to 0.88) with reference PM infection ratings at the leaf image level. The high quantification accuracy of the saliency-based pipeline led to the increased explanation of phenotypic variance and reliable identification of quantitative trait loci. Therefore, the saliency-based processing pipeline can be used as an effective and efficient analysis tool for PM disease research and breeding programs in the future, especially agricultural and life science studies requiring microscopic image analysis.

## Introduction

Grapes are prominent in the North American (NA) agricultural economy [1]. Plant diseases are great challenges to grape production systems, which can dramatically reduce grape productivity and quality and ultimately the profitability of grape and wine industries. Powdery mildew (PM) is a fungal disease that has become a major threat to the grape industry worldwide. In particular, the most common grape cultivars (*Vitis vinifera*) in NA are highly susceptible to the native fungus (*Erysiphe necator*) of grapevine PM, resulting in a significant cost in vineyard PM fungicide applications annually [2]. The intensive fungicide uses also presents concerns about negative environmental impacts. Breeding grape cultivars with PM resistance would be crucial for the long-term productivity, quality, and sustainability of the grape and wine industries.

Recent advancement in genotyping and genetic technologies (e.g. next-generation sequencing, marker assisted selection, genomic selection) have paved the way for effective selection of disease-resistant genotypes, but phenotyping has been widely recognized as a bottleneck to bridge phenomes and genomes to yield new cultivars with desired traits such as disease resistance [3–5]. Human field scouting and manual microscope observation (e.g. hyphal transect counting [6]) are major approaches to evaluating grapevine PM infection in most research studies. Recently, image-based sensing systems have been developed for high throughput plant phenotyping (HTP) of plant stresses, including RGB imaging [7–11], hyperspectral imaging [12], thermal infrared imaging [13], and fluorescence imaging [14]. Some technologies have been particularly used to assess grapevine PM infection [15]. Ultraviolet (UV)-induced fluorescent imaging can reveal PM colonization on grapevine leaves, especially with the ratio of images at 440 nm and 520 nm. Combining an edge detection-based algorithm, grapevine PM colonization could be quantified at 3 days after inoculation (DAI) [16]. This technique has been limited because of potential safety concerns of UV exposure to operators and overdose of UV illumination. Since PM is identifiable in visible light, RGB imaging has become a preferred sensing modality because of its affordability, high spatial resolution, and rich color and texture information [17]. Despite their importance to PM research, all aforementioned studies did not address the need for resolving PM presence and development at the microscopic level, which has been proved effective for genetic studies of grapevine PM resistance [6]. A pioneering study reported the development of a microscopic imaging robot that provided a spatial resolution of 1.2 μm per pixel capable of optically resolving grape PM hyphal without staining [7]. While the robot significantly improved the throughput of image acquisition, image analysis was still a key bottleneck. In particular, the accurate quantification of grape PM infection and development at the microscopic level determines successive quantitative genetics analyses and breeding programs.

Deep learning (DL) techniques such as convolutional neural networks (CNNs) have achieved state-of-the-art performance in computer vision tasks, providing potential solutions to the phenotyping bottleneck. Given many successful uses of deep CNNs to classify images containing stressed plants or plant tissues with high classification accuracy (over 90%) [18–20], a straightforward idea is to combine a CNN classification model with a sampling method (e.g. sliding window) for the quantification of traits of interest [7,21]. An input image can be segregated into multiple subimages for classification, and the number of subimages belonging to each class could be used to calculate quantitative metrics of a phenotype such as disease infection severity (ratio of infected and total subimages). However, there are two major concerns. First, classification models cannot differentiate infection severity within subimages (i.e. all pixels in an infected subimage would be counted as infected), which considerably affects the accuracy of infection quantification. Low phenotyping accuracy might negatively influence the accuracy of quantitative genetics analyses and thus breeding programs. Second, most DL classification models work as a blackbox and provide classification results without explanations or supporting evidence, presenting possible concerns about model validity and generalizability especially for unseen datasets. To overcome and/or mitigate the two issues, processing pipelines based on deep CNN models for object detection and image segmentation have been developed [22,23]. Although detected bounding boxes or segmented masks would provide more accurate quantification of infection, training such models requires a large amount of annotated training samples. Annotating microscopic images with bounding boxes and masks is laborious and costly, especially for disease/pathogen-related datasets, because of necessary domain knowledge and high image spatial resolution. Therefore, it is urgent to develop methods for disease infection quantification at the microscopic level with 1) affordable data annotation requirements, 2) improved quantification accuracy, and 3) human-interpretable visualization. Explainable artificial intelligence (XAI) recently used for the understanding of deep CNN classification models can potentially fulfill all three needs [24–26], because information supporting classification decisions could be generated at the pixel level and used to simultaneously explain the model mechanism and quantify specific infection regions in an image without expensive object−/pixel-level annotations. This motivates the development and investigation of XAI-based approaches for the quantification of disease infection.

Based on the application stage, XAI methods can be categorized as ante-hoc (during model training) or post-hoc (after model training). Due to its easy deployment and unnecessary model retraining, post-hoc XAI methods have been widely used to understand CNN models. Based on the generated explanation format, XAI methods can be classified as visual, rule-based, textual, numerical, or a combination of all. Saliency methods provide saliency maps as visual explanations for the inferential process of a CNN classifier. More importantly, saliency maps present pixel-level information that can potentially be used for extracting quantitative traits in images. As a result, post-hoc saliency methods would be optimal for quantification accuracy improvement and model interpretation/explanation. Interpretability and explainability are considered interchangeable in this study despite a community-wide debate [25].

Currently, there are three major categories of post-hoc saliency methods [27]. Perturbation-based methods (e.g. Occlusion [28]) determine the importance of image regions through perturbated classification evaluation of a CNN model by randomly masking out regions in a given image. These methods are usually computationally expensive, preventing their use for complex CNN models and large datasets. Gradient-based methods [29–31] compute the gradient with respect to the input image by backpropagation to generate saliency maps, representing the importance of a pixel using the calculated pixel intensity. Treating gradients as feature cues is efficient but sometimes has artifacts because of the zero gradient or the discontinuities in the gradient. To avoid such a situation, reference-based methods [21,32] have been developed to utilize contribution scores calculated by comparing the activation of each neuron to its “reference activation” to indicate the importance of pixels. Saliency methods have been integrated into HTP systems to diagnose plant diseases and facilitate the understanding of CNN models. A wide variety of saliency methods were examined to visualize plant symptoms and improve the understanding of the mechanism of CNN models for disease diagnosis [33,34]. An explainable framework dedicated to plant diseases that automated the process of plant stress identification, quantification, and explanation was proposed and seen as the state-of-the-art approach in the plant community [21]. A Teacher/Student architecture leveraging the multitask learning strategy was proposed and achieved sharper visualization than existing methods, highlighting the benefit of the student classifier in guiding the entire model to reconstruct more discriminative regions [35]. A recent exploratory study reported a saliency-based paradigm for grape PM quantification and demonstrated promising quantification accuracy and processing efficiency [36]. Some conventional saliency-based methods have demonstrated an average accuracy of 90% for disease infection segmentation [37]. To have a more comprehensive understanding and further adopt the DL saliency-based paradigm for plant disease quantification, it is necessary to conduct experiments to evaluate 1) the usefulness of saliency maps for domain experts to visualize CNN models and phenotypic trait quantification process, 2) the accuracy of saliency-based approaches for disease severity quantification, especially in microscopic images, and 3) potential impacts for disease phenotyping and successive quantitative genetic analyses and breeding programs.

The major contribution of this study was to develop and comprehensively evaluate a saliency-based processing pipeline for the quantification of grape PM infection at the microscopic level and its applications in PM resistance research and breeding (Figure S1). To our knowledge, this was among the first life sciences studies to i) explore the use of saliency methods for disease quantification at the microscopic level, ii) compare the performance with human evaluation and other annotation-expensive methods, and iii) demonstrate impacts to real world problems in genetics analyses and breeding. Specific outcomes of this work were 1) an optimal data processing pipeline for the quantification of grape PM at the microscopic level and 2) the proposed pipeline was confirmed to be effective and reliable for genetics analyses by extensive genomic experiments.

**Figure 1:**
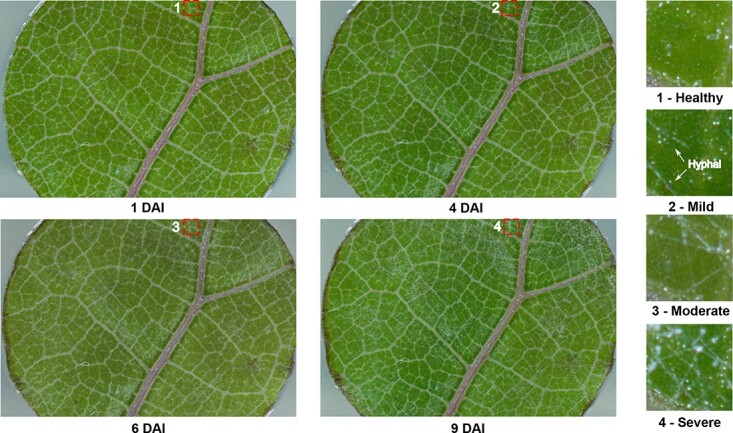
A representative leaf sample imaged at 1, 4, 6, and 9 days after inoculation (DAI) and extracted image patches with different infection severity statuses (healthy, mild, moderate, and severe).

**Table 1:** Summary of datasets used in this study

Dataset	Sample size	Class ratio (healthy versus infected)	Annotation method
DS_CLS	19 362 patches	3:1	Patch class
DS_SEG	1800 patches	3.5:1	Patch class and hyphal mask

The paper was organized as follows. Section 2 describs the biological and computational experiment design, including dataset summary, saliency methods to be evaluated, the workflow of the saliency-based processing pipeline for disease quantification, and experiment details. Section 3 described the sanity check results, CNN model performance, the developed pipeline performance, and the pipeline effects on genetics analyses. Section 4 provids a comprehensive discussion of the whole study. Finally, Section 5 summarizes the conclusions from this study.

**Figure 2:**
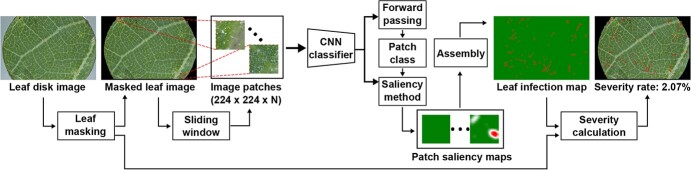
Diagram of the saliency-based processing pipeline for PM infection severity quantification. A leaf sample was masked and cropped to generate valid image patches for classification. A pretrained CNN model classified the generated image patches into healthy or infected categories. Saliency maps of the classified patches were generated to identify pixels determining the classification results (red pixels for infected regions and green pixels for healthy ones). All patch saliency maps were reassembled to construct a leaf-level infection map. The leaf mask and infection map were used to calculate the infection severity of that leaf sample.

## Results

### Sanity check of saliency methods

**Figure 3:**
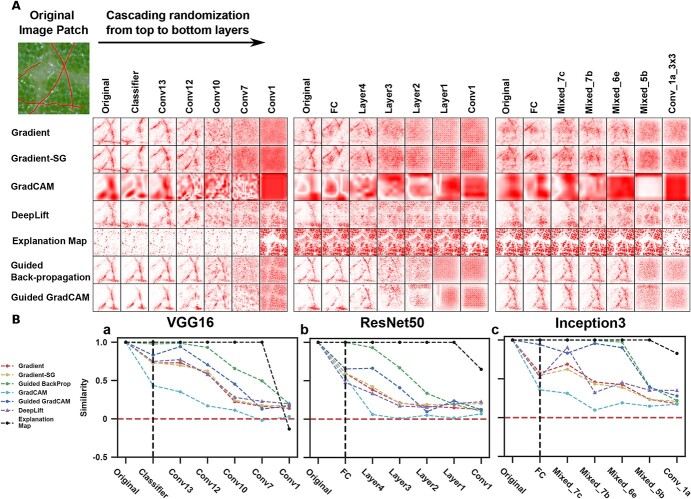
Sanity check results containing saliency maps and similarity measurements. A: Saliency maps generated by VGG16, ResNet50, and Inception V3 with a wide variety of saliency methods. Red pixels were the masks of hyphae in the original image patch. From left to right, saliency maps were calculated for CNN models with weight randomization up to the named layer inclusively. The first column contained results for a fully trained CNN model and the last for results for a completely randomized CNN model. B: Plots of the histogram of gradient (HOG) similarity between saliency maps calculated using a fully trained CNN model and a CNN model with cascading randomization: a VGG16, b ResNet50, and c Inception V3. The vertical black dashed line indicated the start of cascading randomization. The red dashed line indicated the level of zero similarity between the two saliency maps.

Saliency methods (i.e. Gradient, Gradient-SmoothGrad or Gradient-SG, GradCAM, and DeepLift) passed sanity check tests on all three CNN models (Figure 3). When well-trained models were used (the original columns in Figure 3A), these methods identified image pixels of PM hyphae which were the features used by human experts for classification. As soon as model randomization was applied, these methods started to recognize some random pixels that were irrelevant to PM hyphae (Figure 3B). Such a phenomenon became more obvious along with deeper model randomization. This suggested that these methods were training/learning dependent and able to identify pixels truly reflecting key information learned and used by CNN models. Thus, these methods were valid saliency methods and capable of explaining CNN models in classification problems. On the contrary, guided variants (i.e. Guided Back-propagation and Guided GradCAM) and explanation map (EM) failed in sanity check tests irrespective of CNN models. Although the guided variants could generate saliency maps that highlighted PM hyphae pixels with even fewer noises, the generated saliency maps showed almost no difference until some shallow layers of a CNN model were randomized. EM produced saliency maps that did not encode any informative clues in image pixels of PM hyphae and the generated saliency maps showed no difference until the top-most layer was randomized. This means that the guided variants and EM were not truly training/learning dependent and thus saliency maps calculated by these methods could not be used for model explanation. This agreed with a previous study that guided saliency methods intend to do image recovery, not model explanation [47].

It should be noted that EM performed the worst in the sanity check tests and was only able to identify pixels of distinctive background information such as specular spots and leaf veins. In addition, saliency maps generated by EM showed no change unless a model was fully randomized. This occurred primarily for two interrelated reasons. The classification of PM infected image patches in this study was more complex than that of images with various disease symptoms in the original EM study. Infected leaf images in the original EM study showed obvious changes in color (e.g. brownish tissues) and texture (e.g. damaged holes on leaf surface), which could be largely learned in shallow layers of a CNN model. Grapevine PM hyphae were semitransparent lines and looked similar to other features (e.g. leaf hairs) in images in terms of color and texture, requiring features learned by deep layers of a CNN model for representation and classification. The present study adopted the original EM method which used the first convolutional layer to find informative feature maps for the model explanation, resulting in a failure case for interpreting models for PM classification.

In addition to the saliency methods, CNN model architecture showed influences on model explanation (compare the original columns in Figure 3A). When the same valid saliency method was used, saliency maps from VGG16 revealed the most accurate pixels of grape PM hyphae and the least noise pixels, whereas those from Inception V3 and ResNet50 covered much broader infection areas and tended to be noisy. When model randomization was applied, saliency maps from VGG16 showed a smooth decrease in the similarity to the original. In contrast, saliency maps from Inception V3 and RetNet50 showed sharp decreases or kept a high level of similarity (Figure 3B and Figure S2 to Figure S4). This was likely due to the fact that the plain stacking connection in VGG16 would be easier to explain by saliency methods than advanced connection schemes such as Inception modules in Inception V3 and skip connection in ResNet50. Plain stacking connection passed signals between adjacent layers. In contrast, Inception modules and skip connections allowed information to propagate through different pathways or among layers that were at very different model depths, which presented additional challenges for current saliency methods in model explanation.

### Image patch classification and segmentation accuracy

Two core components of the developed saliency-based processing pipeline could be customized, including a CNN classifier and a saliency method. Among the used CNN models, VGG16 provided the marginally highest accuracy (Table S2) and strong generalizability on this series of datasets (Table S3), demonstrating its capability for accurate and robust classification of PM infected image patches. Given better model expandability by the valid saliency methods, VGG16 was selected as the CNN model for successive data analyses in this study. Among valid saliency methods, Gradient-SG and GradCAM were selected because they represented two efficient gradient-based saliency methods using feature maps at two extreme layers (Figure S5).

The segmentation-based pipeline used DeepLab V3 for segmenting infected areas in image patches. DeepLab V3 achieved an average intersection over union (IoU) of 56.27% and Dice of 71.98% on the infected class based on 10 repeated tests (Table S4). Considering the limited training sample size (1800 image patches) and segmentation problem complexity, the model accuracy was acceptable. Inaccurate segmentation mainly came from the false positive prediction of PM infection because of either specular reflectance or a significant color-change caused by necrosis.

### Performance evaluation and comparison of the saliency-based pipeline

#### Quantification accuracy at the image patch level

**Figure 4:**
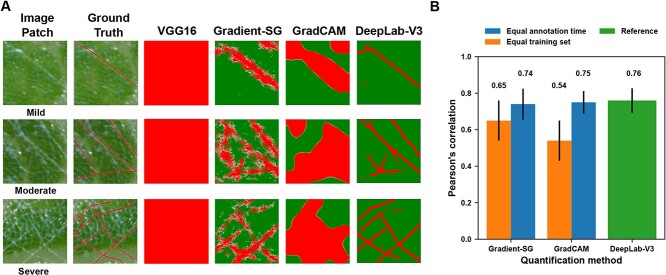
PM quantification results and performance at the image patch level. A: Raw image patches, human annotations, and patch infection maps from classification-based (i.e. VGG16), saliency-based (i.e. Gradient-SG and GradCAM), and segmentation-based (i.e. DeepLab-V3) methods. B: Mean and standard deviation of Pearson’s correlation coefficients between estimated infection severity and the number of hyphal pixels for each quantification method via 10 repeated tests at the image patch level. The quantity on top of each bar indicates the particular correlation coefficient value.

At the image patch level, saliency maps generated by Gradient-SG and GradCAM could recognize PM infected regions for differentiating infection severity among image patches, which was a substantial improvement over image patch classification (Figure 4A). A simple classification of an image patch into infected or healthy would only provide information about the entire patch: all pixels in that image patch belonged to infected or healthy leaf tissue. As a result, infection severity differences (e.g. a single PM hypha to many PM hyphae) among infected image patches could not be distinguished. In contrast, the generated saliency maps aimed to identify pixels that mostly influenced or determined the classification prediction. For infected image patches, these identified pixels would be expected to reflect PM hyphal, leading to improved PM hyphae localization and quantification.

Compared with image segmentation results (i.e. results from DeepLab V3), the generated saliency maps tended to over-identify pixels of grape PM hyphae, showing broader regions and more noises (Figure 4A). GradCAM suffered from these issues primarily because of the resolution loss during the saliency map calculation. GradCAM used the feature map from the last convolutional layer of a CNN model for saliency map computation and upsampled the saliency map to the same spatial resolution of the input image. Depending on CNN model architecture, commonly used deep CNN models considerably reduced the spatial resolution of the output from the last convolutional layer, such as VGG16 for 32 times, which led to the identification of broader PM infection regions. Gradient variants (e.g. Gradient and Gradient-SG) were less problematic with considerable over-identification of PM infection pixels because they propagated gradient values all the way back to the input layer without any spatial sacrifices. These methods yielded many noise pixels due to local feature influences. Although Gradient-SG provided considerably improved results by using Gaussian denoising, the corresponding saliency maps were substantially noisier than that generated by segmentation models such as DeepLab V3. Quantitative analysis results showed that although DeepLab V3 yielded the best correlation with human assessment, such performance gain over GradCAM and Gradient-SG was marginal, especially when different training strategies were used (Figure 4B). When models (i.e. a VGG16 classification model and a DeepLab V3 segmentation model) were trained using the same training set (equal training set), infection severity of image patches measured using segmentation results by the DeepLab V3 model showed a considerably stronger correlation (0.11 to 0.22) with human assessment than that measured using saliency maps from the VGG16 model with GradCAM and Gradient-SG. Annotating an image patch with a class was approximately 10 times faster than that with a hyphal mask, and thus the same annotation time resulted in 10 times more annotated samples for training a VGG16 classification model than a DeepLab V3 model. In this situation, Gradient-SG, GradCAM, and DeepLab V3 showed no significant performance difference (Table S5). A possible reason was that deep CNN models would benefit from a large training dataset to learn robust features for a particular problem, which assisted in the accurate identification of relevant pixels in saliency maps. Therefore, although saliency methods could not be used to replace segmentation models in accurate mask generation, they offered an improved way to quantification of PM infection severity at the patch level.

#### Quantification accuracy at the leaf sample level

**Figure 5:**
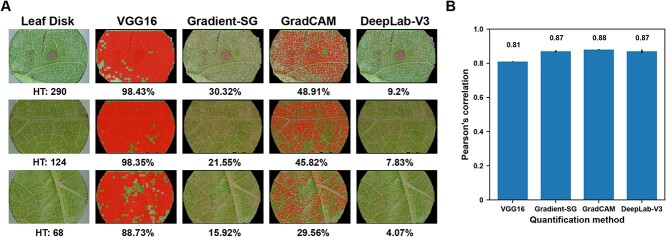
PM quantification results and performance at the leaf sample level. A: Raw leaf samples, samples overlaid with infection maps generated by the classification-based (i.e. VGG16), saliency-based (i.e. Gradient-SG and GradCAM), and segmentation-based (i.e. DeepLab-V3) pipelines. Hyphal transect counts (i.e. HT) and estimated infection severity were provided below each raw leaf sample and overlaid images, respectively. B: Mean and standard deviation of Pearson’s correlation coefficients between estimated infection severity and hyphal counts for each processing pipeline via 10 repeated tests at the leaf sample level. The quantity on top of the bar indicates the particular correlation coefficient value. CLS means the classification-based pipeline, SAL-SG/GradCAM means the saliency-based pipeline with Gradient-SG and GradCAM, and SEG means the segmentation-based pipeline.

At the leaf sample level, three processing pipelines were evaluated and compared for infection severity calculation, including an image patch classification-based pipeline, an image patch saliency map-based pipeline, and an image patch segmentation-based pipeline. For brevity, the three pipelines were referred to as classification-based, saliency-based, and segmentation-based pipelines. Representative results showed that the three types of processing pipelines had different patterns in the quantification of grape PM infection in full leaf sample images (Figure 5A). The classification-based pipeline provided the highest measurement magnitude of PM infection severity and tended to dramatically over-estimate the infection. For instance, infection severity calculated by the classification-based pipeline could exceed 98%, meaning that almost the entire leaf sample surface was occupied with PM hyphae. This deviated from the fact that PM hyphae would not develop and occupy such a large area on a leaf sample through the course of the experiment based on biological principles. Furthermore, such high measurement magnitudes limited the capability of representing different levels of leaf infection severity (compare the hyphal transect counts and classification-based results in Figure 5A and Table S6). In contrast, the saliency- and segmentation-based pipelines obtained measurement ranges much closer to empirical observations and kept distinguishable relative differences of infection severity among leaf samples, especially when infection occurred with different patterns. The three representative leaf samples had substantial relative differences (approximately 50%) in hyphal counts using the hyphal transect protocol (from top to bottom in Figure 5A). The classification-based pipeline identified relative severity differences of 0.08% and 10% between the top and middle and middle and bottom samples, respectively, whereas the saliency- and segmentation-based pipelines provided much closer estimations due to their finer localization and quantification of PM infection (Table S6).

When the best CNN model was used, saliency-based processing pipelines showed significant improvements in quantification accuracy: the Gradient-SG and GradCAM-based saliency pipelines achieved 7% and 6% higher correlations with hyphal transect counts than that of the classification-based pipeline (Figure 5B and Table S7 and S8). This demonstrated a substantial improvement of the saliency-based pipelines for the quantification of PM infection severity at the leaf sample level. Particularly, the performance of Gradient-SG was slightly better (1%) than that of the segmentation-based pipeline (segmentation-based was equivalent to GradCAM-based), meaning that the saliency-based pipeline could be a valid alternative to the segmentation-based pipeline for disease infection quantification. It was noteworthy that the correlation between hyphal transect count and human image rating was 0.88, so both the saliency- and segmentation-based pipelines reached the human performance level (Figure S6). These suggested that the saliency-based pipeline (either with Gradient-SG or GradCAM) was an effective (in terms of the quantification accuracy) and efficient (in terms of the annotation complexity and cost) approach for PM infection quantification. The segmentation-based pipeline was not further evaluated for two reasons: first, the present study primarily investigated the efficacy of the saliency-based pipeline. Second, the training dataset for the segmentation model (e.g. DS_SEG) contains 1440 samples, which could be a limiting factor for model generalizability. Thus, only the classification- and saliency-based pipelines were compared in successive analyses.

#### Phenotypic traits for PM infection

**Figure 6:**
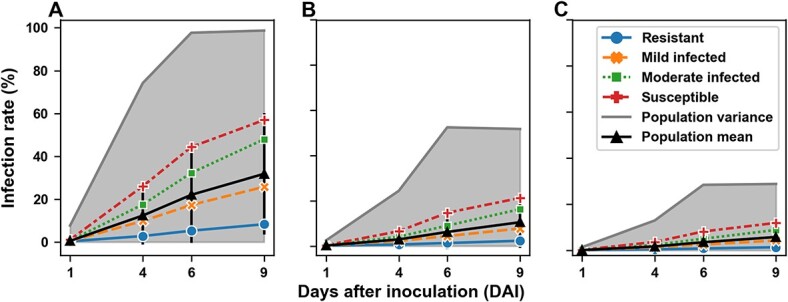
Infection rates as a function of the day after inoculation (DAI) for four different genotypes. A: Classification-based pipeline. B: Saliency-based pipeline with GradCAM. C: Saliency-based pipeline with Gradient-SG. The infection rate of a genotype was computed as the mean of the infection rate of all samples belonging to this specific genotype.

Generally, the classification- and saliency-based pipelines showed similar sigmoidal PM infection development patterns from 1 DAI to 9 DAI due to an exponential increase in pathogen growth at the early stage followed by saturation of available host tissue later (Figure 6). The mean infection severity rates of the whole population and four representative genotypes (i.e. resistant, mildly infected, moderately infected, and susceptible) gradually increased over the time of the experiment. Experimental results well agreed well with the genotype information that the resistant genotype showed the least infection followed by mildly infected, moderately infected, and susceptible genotypes. A major difference between the classification- and saliency-based pipelines was the infection development rates from 1 DAI to 4 DAI and from 4 DAI to 6 DAI. The classification-based pipeline revealed a faster PM development from 1 DAI to 4 DAI than that from 4 to 6 DAI, whereas the saliency-based pipeline (regardless of saliency methods) reflected an opposite pattern. Grapevine PM hyphae typically appeared from 1 DAI to 4 DAI and considerably expanded from 4 DAI to 6 DAI while new hyphae kept establishing. The classification-based pipeline was not able to differentiate the infection severity of individual image patches, so PM hyphal development would barely affect the severity quantification, resulting in a limited change of infection severity increasing the rate from 4 DAI to 6 DAI. On the contrary, the saliency-based pipeline was able to distinguish PM hyphal emerging and growing stages, showing increases in infection severity rate from 4 DAI to 6 DAI. Although the population variance showed a plateau after 6 DAI, the mean infection rates of the representative individual genotypes kept increasing, which implied that phenotypic traits after 6 DAI might be necessary for genetic analyses.

**Figure 7:**
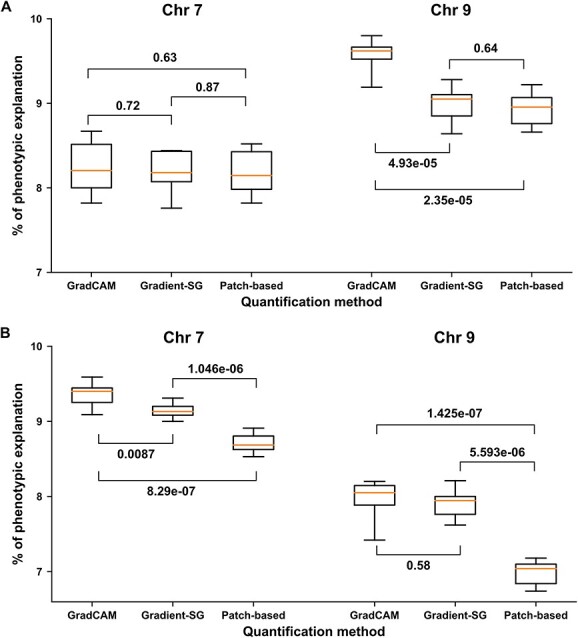
The phenotypic variance explained by chromosomes (Chr) 7 and 9 for phenotypic traits calculated by the classification-based and saliency-based processing pipelines. Quantity across two methods is the p value from t-tests. A: Results based on the most effective trait, infection severity on 4 DAI data (i.e. IS4). B: Results based on the accuracy-sensitive trait, mean infection severity over the course of the experiment (i.e. mean IS).

### Effects on genetic analysis

#### Phenotyping accuracy effects

Based on a preliminary QTL mapping analysis in this study, seven selected phenotypic traits could be categorized into three groups: traits that enabled the identification of two QTLs regardless of measurement methods (defined as most-effective traits); traits that enabled the identification of two QTLs when quantification accuracy was improved (defined as accuracy-sensitive traits); and traits that revealed only one QTL regardless of measurement methods (defined as least-effective traits) (Table S9). Infection severity on 4 DAI (IS4) was the only most-effective trait in this study, enabling the identification of both QTLs regardless of the measurement method. The mean infection severity over the course of the experiment (mean IS) was selected to represent the accuracy-sensitive traits in further analyses, though IS6 performed similarly well (Table S9). The two traits calculated using the classification- and saliency-based pipelines were used to examine the effects on genetic analyses in detail (Figure 5).

The saliency-based pipeline demonstrated reliably better performance in QTL mapping analysis than the classification-based pipeline using either the most effective or accuracy-sensitive trait (Figure 5). In an ideal situation where the most effective trait, IS4, was used, all three approaches provided highly comparable explanations of phenotypic variance for chromosome 7, and the GradCAM-based saliency pipeline explained statistically more phenotypic variance for chromosome 9 than the Gradient-SG and classification-based pipelines (Figure 5A). Nonetheless, all the explained phenotypic variances exceeded the significance threshold in QTL mapping, so the quantification accuracy improvement through the saliency-based pipeline did not lead to the identification of new QTLs in genetic analyses when a highly effective trait was used (Table S10). In the accuracy-sensitive case (mean IS), the GradCAM and Gradient-SG based saliency pipelines provided significantly better explanations of the phenotypic variance than the classification-based pipeline. In particular, the explained phenotypic variances from the classification-based pipeline failed to pass the significance threshold, resulting in missing the QTL on chromosome 9, whereas the explained variances from the saliency-based pipelines (both GradCAM and Gradient-SG based) consistently exceeded the significance threshold (Table S11). This demonstrated that the quantification accuracy improvement of the saliency-based pipeline could dramatically improve the stability of QTL mapping analysis to minimize potential negative effects due to the sensitivity of quantification accuracy for a particular phenotypic trait.

#### Model training effects

The use of training samples could considerably affect CNN model performance in image patch classification and ultimately the quantification accuracy of corresponding processing pipelines. In the experiments, different partitioning seeds of the training dataset showed no statistically significant influence on the quantity of the phenotypic variance explained by different processing pipelines (Table S12 and S13). When the significance of the explained phenotypic variances was considered, the classification- and saliency-based pipelines achieved dramatically different performances in the QTL mapping analyses (Table 2). With the most-effective trait (IS4 in this study), all pipelines consistently revealed significant phenotypic variances and were able to successfully identify QTLs on chromosomes 7 and 9, whereas with the accuracy-sensitive trait (mean IS in this study), the classification-based pipeline provided insignificant phenotypic variances and failed to identify QTLs on chromosome 9. On the contrary, both the GradCAM and Gradient-SG based saliency pipelines yielded significant phenotypic variances and revealed QTLs with a high success rate (> 80%) with various training data partitioning seeds and ratios. This further confirmed that the saliency-based pipelines were accurate and robust with training sample selection. It should be noted that the GradCAM-based pipeline steadily explained more phenotypic variances than the Gradient-SG based one. Although no significant improvement or new QTLs were identified in this study, such a higher explained phenotypic variance might lead to new findings in other experiments. This is worthy of further investigation in the future.

**Table 2:** Mean and standard deviation of percent of the explained phenotypic variance as well as the percentage when the genetic locus is statistically significant at α = 0.05 for the particular quantification method

Method	Varied partitioning seeds				Varied partitioning ratios			
	Most effective trait (IS4)		Accuracy sensitive trait (mean IS)		Most effective trait (IS4)		Accuracy sensitive trait (mean IS)	
	Chr 7	Chr 9	Chr 7	Chr 9	Chr 7	Chr 9	Chr 7	**Chr 9**
Classification-based pipeline	8.17 ±0.26 *(100%)*	8.93 ± 0.20 *(100%)*	8.71 ± 0.12 *(100%)*	**6.98 ±** **0.16** ***(0%)***	8.26 ± 0.23 *(100%)*	8.98 ± 0.09 *(100%)*	8.68 ± 0.11 *(100%)*	**6.94 ± 0.11 *(0%)***
GradCAM-based saliency pipeline	8.25 ±0.31 *(100%)*	9.57 ± 0.18 *(100%)*	9.36 ± 0.16 *(100%)*	**7.97 ±** **0.24 *(87.5%)***	8.38 ± 0.41 *(100%)*	9.62 ± 0.18 *(100%)*	9.33 ± 0.28 *(100%)*	**7.93 ± 0.19 *(80%)***
Gradient-SG based saliency pipeline	8.19 ±0.23 *(100%)*	8.99 ± 0.20 *(100%)*	9.14 ± 0.10 *(100%)*	**7.90 ±** **0.19 *(87.5%)***	8.29 ± 0.27 *(100%)*	8.99 ± 0.19 *(100%)*	9.18 ± 0.13 *(100%)*	**7.78 ± 0.20 *(80%)***

## Discussion

Extensive experiments in this study confirmed that the developed saliency-based image processing pipeline can improve the quantification accuracy of PM infection severity (therefore phenotyping accuracy) and ultimately the reliability and performance of genetic analyses such as QTL mapping. This agrees with our previous exploratory work [36]. An important part of the saliency-based pipeline is the validity of saliency methods. Some studies have argued that saliency methods are not valid for two reasons [48, 49]: first, it is unclear if saliency methods truly reveal images pixels determining classification prediction, which has been addressed by the sanity check tests. Valid saliency methods (e.g. Gradient-based variants and DeepLift) were identified as those being able to show the effectiveness of identifying pixels/pixel regions determining the classification prediction of CNN models. However, some classical saliency methods (e.g. Guided backpropagation and guided GradCAM) are essential for image recovery rather than the actual saliency of importance to model inference process [47], so they are not suitable for saliency-based quantification of PM infection or other similar applications. These findings agree with other previous studies [38]. It should be noted that the invalidity of the EM method is largely due to the use of the first convolutional layer for feature importance calculation. For complex problems, one would expect to use deep layers rather than shallow ones to ensure the recognition of key representations for complex concepts. Second, although saliency methods can identify valid pixels, there is an argument on the usefulness of the identified pixels because these pixels simply reflect locations to be used by CNN models rather than meaningful explanation. In fact, the soundness of such an argument highly depends on applications. Although it might be important to explain all key components in an image for classification, revealing locations would be sufficient for the understanding of CNN models for PM identification or disease identification in general. This is because localizing infection symptoms is the most critical step human experts would perform, especially for differentiating healthy and infected samples. On the other hand, having the capability to further explain whether a particular appearance (e.g. color, texture, etc.) leads to a prediction result would be extremely valuable. This is worth exploring in the future.

One key advantage of the saliency-based pipeline is the use of post-hoc saliency methods, which offer great flexibility in integrating a CNN classification model for the quantification of a trait in a wide range of potential applications. Therefore, many CNN classification models from previous and ongoing projects could be revisited and reused for conducting quantitative biological analyses (e.g. QTL mapping) without extra efforts on data annotation or model retraining. This may help scientific communities to mine additional information from historical experimental data. Another key advantage of the saliency-based pipeline is the enhanced understanding of deep learning models used in research studies. It is widely recognized that deep networks are likely to have overfitting issues because of their large number of learnable parameters. As an intermediate result of the saliency-based pipeline, the saliency map of an input image could help human researchers to examine the meaningfulness of features learned and used by CNN models for a given task (e.g. PM hyphae in this study) and to guide further improvements or model tuning. Both would help to build the trust and confidence in adopting deep learning techniques for plant science research and breeding programs that are crucial to food and nutrition security worldwide. Lastly, the saliency-based pipeline relies on CNN classification models for quantification. Annotating image classes requires much simpler operations and less domain knowledge for human labelers than annotating image masks for segmentation. The same annotation expense (e.g. time and cost) in image class annotation would yield more annotated training samples than that in image mask annotation, which will lead to improved performance and robustness of trained models. Therefore, the saliency-based pipeline is an effective, efficient, and reliable approach for the quantification of grape PM infection.

While the saliency-based pipeline achieved comparable quantification accuracy, it has several limitations in the present study. The saliency-based pipeline could not replace the segmentation-based one for several challenges. First, the classification accuracy should be further improved because only correctly predicted infected image patches will be post-processed by saliency methods. Since the CNN classifiers achieved approximately 93% accuracy on testing images, it is reasonable to expect an additional 2 to 4% improvement by using advanced CNN model architecture and training techniques. Second, current valid saliency methods suffer from issues such as noisy results (e.g. results from Gradient-SG) and spatial resolution reduction (e.g. results from GradCAM). These two issues come from the same challenge: how to properly transfer the feature importance/influence on classification back to the input layer of a CNN model. Direct propagation of gradients might not be an optimal solution,because some features (eventual pixels at the input layer) could have a strong global influence but a small local derivative, leading to noisy results [31]. Feature maps from deep layers (e.g. the last convolution layer used by GradCAM) have significant spatial resolution reduction because of stride and pooling operations. Simple upsampling of the importance from such deep feature maps naturally causes reduced spatial resolution of saliency results. As the XAI community is growing intensively, many newly developed post-hoc saliency methods are emerging and can be applied to the saliency-based pipeline. If saliency methods can resolve finer spatial details, the quantification accuracy of the saliency-based pipeline would further improve. Such improvements might open new opportunities for using the saliency-based pipeline for image segmentation.

For genetic analyses, phenotyping accuracy could have a significant impact on results, especially for traits that are sensitive to quantification accuracy. The QTL mapping analyses in this study aimed to find QTLs related to PM infection resistance and used various phenotypic traits for infection severity. The use of an informative trait (e.g. IS4 in this study) measured by a low-accuracy method (e.g. the classification-based pipeline) could identify the same QTLs that are detected using that trait measured by a high-accuracy method (e.g. the saliency-based pipeline). Therefore, one may need to derive and test phenotypic traits that can be potentially measured in a given dataset for not missing QTLs. On the other hand, improving phenotyping accuracy is crucial for genetics/genomics analyses. In particular, with a high quantification accuracy, different phenotypic traits measured by the saliency-based pipeline could be used to reliably reveal the same QTLs. This could help to minimize or even fully eliminate potential risks of missing QTLs by using an ineffective phenotypic trait. Both findings suggest that reliable genetics/genomics analyses essentially require both high accuracy and throughput for phenotyping traits of interest.

## Conclusion

This study developed and evaluated a saliency-based paradigm that integrates saliency methods with CNN classifiers for the quantification of grapevine PM infection severity at the microscopic level. PM infection traits extracted by the saliency-based processing method achieved a strong correlation (r = 0.88) with PM hyphal counts by an established protocol. This performance was significantly better than a classification-based processing pipeline and was comparable with segmentation-based methods and manual image-based counting. Such quantification accuracy improvements also led to increased stability of identifying QTLs in the QTL mapping analyses, especially for traits sensitive to quantification accuracy. Additionally, generated saliency maps could be used to understand the inferential process of CNN models by human experts, enhancing the confidence in adopting deep learning models for practical applications. Therefore, the saliency-based processing method is effective, efficient, and trustworthy for grapevine PM infection quantification at the microscopic level. Future studies will focus on the application of the saliency-based pipeline for other mapping families in genetics studies and breeding programs, and the improvement of saliency map accuracy for the identification and localization of plant stresses.

## Materials and methods

### Experimental design and data acquisition

A total of 2690 leaf samples (267 progeny and 2 check vines with each of 10 replicates) were collected from a biparental grapevine mapping family NY84.0101.03 × *V. rupestris “*Pillans*”* known to be segregating for PM resistance. The samples were prepared using a leaf disk assay and imaged to generate a dataset consisting of 10 760 leaf disk images (8256 × 5504 pixels). A total of 21 162 image patches (224 × 224 pixels) were randomly selected from the leak disk images for model training, validation, and testing. More details of biological background, sample preparation, and image collection were reported in our previous work [6,36]. All the image patches were labeled with an infection class (i.e. healthy or infected), and the same amount of time on class labeling for DS_CLS was used to annotate hyphal masks for DS_SEG (Table 1). DS_CLS and DS_SEG were from different leaf samples, so they could be used as independent testing sets for each other. Hyphal transect, an established protocol [6], was used to measure the reference PM infection rate for additional 102 leaf samples from a separate experiment for testing purposes. The protocol uses a point-intercept method and counts the number of hyphal across the horizontal and vertical sections of a leaf sample [6].

### Sanity check of saliency map methods

Saliency maps (or pixel attribution) are grayscale images to record and visualize the importance of individual pixels to the classification decision-making [24]. A previous study demonstrated that some saliency methods might not truly reflect pixel importance for model inferential processes, so it was necessary to conduct sanity check tests to identify valid saliency methods for use [38]. An approach of model randomization was adopted from that study [38] to examine the explanation/interpretation capability of saliency methods. In a model randomization experiment, a saliency method was used to generate an original saliency map from a well-trained CNN model as a reference. Learned weights of the well-trained model were randomized successively from the top (output) to bottom (input) layers. Meanwhile, the same saliency method was used to compute a saliency map from each of the randomized models. The resultant saliency maps were compared with the reference map by visualization and similarity metrics including Spearman’s rank correlation, Structural Similarity Index (SSIM), and histogram of gradient (HOG). A valid saliency method should be model learning dependent, meaning it is expected to show a continuous reduction in the similarity between the reference and randomized-model saliency maps. Among different CNN models, differences of saliency maps generated by the same saliency method reflect potential influences of model architecture on model explanation. In this study, commonly used saliency methods were tested including gradient-based methods (conventional Gradient [29], Gradient-SmoothGrad or Gradient-SG [31], GradCAM [30]), guided-gradient variants (Guided Back-propagation [39] and Guided GradCAM), and two reference-based methods (DeepLift [32] and Explanation Map [21]). Valid saliency methods were used to develop a saliency-based processing pipeline for the quantification of PM infection.

### Saliency-based pipeline for the quantification of PM infection

A saliency-based pipeline was developed for the quantification of PM infection at the microscopic level (Figure 2). There were four modules in the processing pipeline including 1) leaf masking, 2) leaf image segregation, 3) image patch PM identification, and 4) leaf PM quantification. A leaf image was processed by the leaf masking module to remove background information around the leaf sample. Intuitively, the leaf mask algorithm used variance-based image sharpness to filter out 1) sample tray areas with mostly uniform color and little to no texture and 2) defocused leaf regions (more details were provided in the Leaf Masking section in the supplementary materials). The resultant image was segregated into image patches for processing. In the present study, image patch size was defined as 224 × 224 pixels. Through the image patch PM quantification module, the image patches were classified by a pretrained CNN classifier. Saliency maps were generated by a saliency method for infected patches, and a blank saliency map (pixel intensity was zero) was assigned to healthy patches, showing no influence on the classification of PM infection. The patch saliency maps were reassembled into leaf infection maps for calculating the PM infection severity of individual leaf images. The CNN model, saliency method, and thresholding strategy were key components to be optimized in this pipeline. Previous experiments showed that an arbitrary threshold of 0.2 was optimal for yielding the best quantification accuracy at the patch and leaf disk levels [36], so the threshold was adopted for the experiments in this study. The optimal configuration of CNN models and saliency methods were comprehensively examined in this study.

### Quantification performance evaluation and comparison

#### Classification and segmentation performance on image patches

Three representative CNN classifiers (VGG16 [40], ResNet50 [41], and Inception V3 [42]) were used for image patch classification because of their distinctive, widely-adopted model architecture (i.e. plain stacking via VGG16, skip-connection via ResNet50, and inception module for wide network via Inception V3) and high classification performance. Models were trained and validated using the DS_CLS dataset with a training/validation ratio of 8:2 and tested using the DS_SEG dataset. Classification accuracy and F1 score on the validation and testing sets were indicators for the evaluation of model performance. Since the classification training and validation sets were partitioned from a dataset containing samples imaged at four different DAIs, features in the validation set may have already appeared in the training set. To exclude the potential artifacts brought up by the mixed data and rigorously validate the generalizability of CNN models, cross validation in terms of DAI was conducted. Samples imaged at a particular DAI were treated as the validation set while samples imaged at another three DAIs were used to train the model (Table S1).

As deep learning-based segmentation models provided state-of-the-art performance in disease quantification [22], a commonly used model, DeepLab V3 [43], was trained and tested for PM hyphal segmentation in image patches using the DS_SEG dataset with a training/testing ratio of 8:2. ResNet101 [41] was used as the backbone feature extractor of DeepLab V3. IoU and Dice scores for the infected class were used to evaluate the segmentation performance because of a highly imbalanced class distribution where the average IoU and Dice scores could be dominated by the healthy instances. It should be noted that PM hyphal lines were labeled using polylines with a width of 1 pixel, presenting challenges for segmentation model training and evaluation. To overcome this issue, hyphal masks in the DS_SEG dataset were thickened by preprocessing using a 5 × 5 dilation kernel with OpenCV (v4.2.0) for segmentation. The kernel size was determined in a way so that the dilated hyphae in digital images have the approximately same width as the hyphae in physical leaves.

Transfer learning was used to improve training efficiency and effectiveness for both classification and segmentation tasks. In this study, all models were pretrained on a large-scale dataset (i.e. the ImageNet dataset for classification or a subset of COCO train2017, which contains 20 categories that are present in the Pascal VOC dataset for segmentation) and fine-tuned on the training set. On-the-fly data augmentation was applied to increase the diversity of training samples, including horizontal and vertical flipping for both tasks with additional image rotation, translation, and scaling for classification. The Adam optimizer was used for model training with an initial learning rate of 10^−4^, weight decay of 2 × 10^−4^, and a batch size of 100 or 32 for classification and segmentation, respectively. All models and computing programs were implemented using PyTorch (v1.7, Facebook’s AI Research Lab) and performed on a GPU server with 20 CPU cores (3.7 GHz per core), 128 GB of system memory, and a dedicated GPU card (NVIDIA RTX Titan 24 GB). Models were finetuned for a total of 300 and 100 epochs for classification and segmentation, respectively. The checkpoint of each model with the best accuracy on the validation set was selected for successive analyses.

#### Quantification accuracy at the image patch level

A key component of the saliency-based processing pipeline was to quantify PM infection of image patches by combining CNN classifiers and saliency methods, so it was important to evaluate the quantification accuracy at the image patch level. Two experiments were conducted to compare patch PM infection severity from saliency maps with those from segmentation results and human annotations. The first experiment was for the performance comparison when the same training dataset was used, which is considered fair from the data science perspective. DS_SEG was partitioned into training and testing sets. The training set was used to train CNN classification and segmentation (DeepLab V3) models. The testing set was processed by using optimal combinations of CNN classifier and saliency method to generate saliency maps and using the trained DeepLab V3 model to generate hyphal masks, respectively. Resultant saliency maps were binarized using the same threshold in the saliency-based pipeline. The number of pixels determining infection (namely belonging to PM hyphae in this study) was calculated from the binarized saliency maps and hyphal masks as the metric to quantify the PM infection severity of individual image patches. PM hyphae were considerably thin in images, so the localization of PM hyphae was visually compared among the binarized saliency maps, hyphal masks, and human annotations. Pearson’s correlation analyses were conducted between the number of hyphal pixels from human annotations and that from image-derived results (either saliency maps or hyphal masks). Pearson’s correlation coefficient (r) was used to indicate the performance. The second experiment was a performance comparison when the same annotation effort (i.e. time) was made, which is more meaningful in practical applications. All processing procedures were the same as in the first experiment with the exception that CNN classifiers were trained using a training set randomly selected from DS_CLS, which was annotated using the same human time but contained approximately 10 times more images than DS_SEG. This would reveal the potential benefits of using saliency-based approaches for quantification in real world setups. Each of the two experiments was conducted repeatedly for 10 times by setting a different random seed for data partitioning to avoid potential effects caused by training sample selection. Analysis of variance (ANOVA) tests were conducted to reveal the statistical performance differences at a significance level of 0.05.

#### Quantification accuracy at the leaf sample level

The ultimate goal was to quantify PM infection for individual leaf samples, so the quantification performance at the leaf image level was evaluated. The best combination of CNN classifier and saliency method from the patch-level experiments was used as the components in the saliency-based processing pipeline. For comparison purposes, two additional PM quantification processing pipelines were used: one classification-based and one segmentation-based. The key difference among the three processing pipelines was the patch-level PM quantification. The classification-based pipeline counted all pixels of an infected patch, whereas the saliency-based and segmentation-based pipelines relied on saliency maps and hyphal masks, respectively, for the quantification at the patch level. The classification-based processing pipeline was developed for the original PMBot data analysis [7] and treated as the baseline in comparisons. The segmentation pipeline was considered to provide state-of-the-art accuracy. All pipelines were implemented using PyTorch (v1.7) to avoid potential performance artifacts due to differences in programming languages and deep learning libraries in the present study. The 102 leaf samples with reference PM infection rates were used to evaluate the quantification accuracy of the classification-, saliency-, and segmentation-based pipelines. Pearson’s correlation analyses were conducted between PM infection rates calculated by the three pipelines and hyphal transect counts by human experts, separately. Pearson’s correlation coefficient (r) was used to indicate the performance of PM infection quantification for the three pipelines. The experiment was conducted repeatedly for 10 times to avoid potential performance fluctuations caused by random training sample selection. Analysis of variance (ANOVA) tests were conducted to reveal the statistical performance differences at a significance level of 0.05.

#### Extraction of phenotypic traits for PM infection

A total of seven traits were extracted from each leaf sample image, including four describing infection status and three for infection dynamics over the course of the experiment. The four status traits were PM infection severity at 4 (IS4), 6 (IS6), and 9 (IS9) days after inoculation (DAI) and average PM infection severity (mean IS) over all time points. The three dynamic traits were the area under the disease progress stairs (AUDPS), infection growth rates between 4 and 6 DAI (IG4–6), and 6 and 9 DAI (IG6–9), respectively. In addition to the traits for individual leaf samples, group mean and variation of these traits were calculated both for the entire population and for four genotypes (resistant, mild infected, moderate infected, and susceptible) representing four levels (healthy, mild, moderate, and severe) of PM resistance. The four genotypes were selected based on hyphal transect counts from the 102 leaf samples. Hyphal counts of 0, 10 to 25, 40 to 60, and over 70 were the criteria for selecting the genotypes with the four levels of PM resistance. It should be noted that the segmentation-based processing pipeline was not used to extract phenotypic traits of all sample images because of potential concerns about the generalizability of DeepLab V3 models trained on a small-size dataset.

### Effects of phenotyping performance on genetic analyses

#### Genotyping and QTL mapping analysis

Genotyping and QTL analysis were performed as previously described [6]. Briefly, a genetic map was constructed using Lep-MAP3 v.0.2 software using 2000 core genome local haplotype markers [44,45]. The genetic information from this map was uploaded with the extracted phenotypes into R/qtl and was run as a four-way cross format [46]. A QTL was considered detected if it crossed the significance threshold (α = 0.05) obtained by running 1000 permutations. For the phenotypes with skewed distribution, log transformation was conducted to fulfill the assumptions of the model or was analyzed with a non-parametric method when the assumptions could not be met.

#### Phenotyping accuracy effects

While the accuracy of phenotypic trait extraction would be improved by using the saliency-based processing pipeline, it was not clear if such accuracy improvements could lead to better results or new findings in genetic analyses such as QTL mapping. To investigate this, QTL mapping analyses were performed using the traits extracted by the classification- and saliency-based processing pipelines. Results were noted if 1) new QTL were identified by traits with improved quantification accuracy and 2) the phenotypic variance was better explained. CNN classifiers used in both classification- and saliency-based pipelines were trained using 8 randomly generated training sets, thus the phenotypic traits were extracted from 8 replicate pipelines to show the statistics of the observed results. To avoid exhausting tests on all traits via 8 replicate pipelines, preliminary QTL analyses were conducted to select representative traits for a comprehensive examination.

#### Model training effects

Training dataset partitioning could dramatically affect training sample distribution and thus trained model performance. It was crucial to examine such training performance effects on genetics analyses such as QTL mapping. Data partitioning has two key parameters: the partitioning seed determines how each set is composed and the partitioning ratio determines how much amount of data each set has. Two experiments were conducted. First, with a fixed partitioning ratio (8/2 for training/validation in this study) but eight different partitioning seeds, eight of such training/validation sets were built and then used for training CNN models. Second, five training/validation sets were made from the same procedure but under a different condition, where the partitioning ratio ranged from 5/5 to 9/1 with a step of 1 for the training/validation set and a fixed partitioning seed. All the trained models were integrated with the saliency-based processing pipeline to extract the seven phenotypic traits for QTL mapping. ANOVA tests were performed to check potential statistical differences in QTL analysis results at the significance level of 0.05. All ANOVA tests were conducted using RStudio with R (v4.2.0).

## Supplementary Material

supp_data_uhac187

## Data Availability

The source code and raw data used in this study will be shared with the public upon the acceptance of the manuscript. For review purposes, please check the project GitHub repository https://github.com/suptimq/Saliency_based_Grape_PM_Quantification.
