# Supplementary material for: High throughput saliency-based quantification of grape powdery mildew at the microscopic level for disease resistance breeding
Source: Hortic Res. 2022 Aug 25;9:uhac187. doi: 10.1093/hr/uhac187 (PMC9630970; doi:10.1093/hr/uhac187)
Supplement: supp_data_uhac187 [file supp_data_uhac187.zip › SupplementaryMaterials_Clean.docx]

**High throughput saliency-based quantification of grape powdery mildew at the microscopic level for disease resistance breeding**

# Supplementary Material

Tian Qiu^1^, Anna Underhill^2^, Surya Sapkota^3^, Lance Cadle-Davidson^2, 3^, Yu Jiang^4, *^

^1^School of Electrical and Computer Engineering, College of Engineering, Cornell University, Ithaca, NY 14850, United States of America.

^2^United States Department of Agriculture-Agricultural Research Service, Grape Genetics Research Unit, Geneva, NY 14456, United States of America.

^3^Plant Pathology and Plant-Microbe Biology Section, School of Integrative Plant Science, Cornell University, Geneva, NY 14456, United States of America.

^4^Horticulture Section, School of Integrative Plant Science, Cornell University, Geneva, NY 14456, United States of America.

*Corresponding author is Yu Jiang at Cyber-Agricultural Intelligence and Robotics (CAIR) Laboratory, 301 Hedrick Hall, 635 W North St, Cornell AgriTech, Cornell University, Geneva, NY 14456, USA. Email: yj522@cornell.edu. Tel: +1 315 787 2220.

**Leaf Masking**

The leaf masking algorithm iterated over the input image using a sliding window (50×6 pixels in this study). The standard deviation of each color channel was calculated for the image region of the sliding window. The maximum standard deviation of the three channels was used to represent the image sharpness. The sharpness map of the leaf disk image was binarized to produce a coarse leaf mask using a threshold derived from the maximum standard deviation. Based on the current robot configuration, the threshold was 0.8× the maximum standard deviation in this study. The coarse leaf mask was applied to the sharpness map to generate a second threshold (mean value of the masked sharpness map) for a refined leaf mask. The refined leaf mask was further improved via a combination of morphological operations to produce the final leaf mask. The number of the connected components in the final leaf mask was calculated to validate the effectiveness. For sample tray/defocused regions, little to no pixels would be identified in the final leaf mask. Therefore, if the no component was identified or the largest component was less than 15% of the sliding window size, the leaf mask would be invalid.


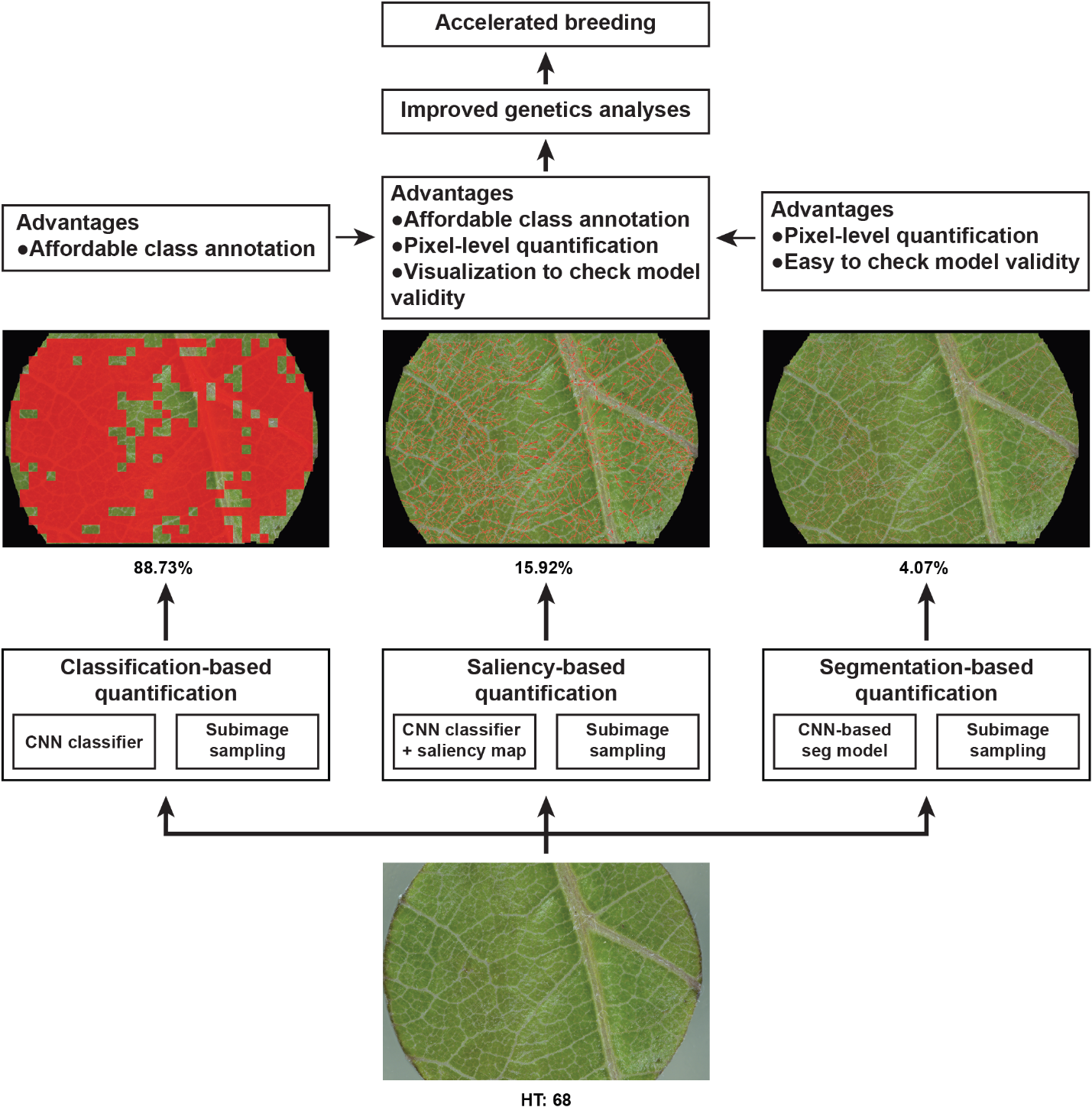


**Figure S1. Schematic overview of the key improvements of the saliency-based processing pipeline for disease quantification at the microscopic level.**

Table S1. Summary of datasets used for cross validation in this study.

| Cross validation on i-th days after inoculation (DAI) | Dataset | Dataset size | Class ratio (healthy versus infected) | Annotation method |
| --- | --- | --- | --- | --- |
| 1 | Training | 16134 patches | 2.21:1 | Patch class |
|  | Validation | 3228 patches | 7 infected only | Patch class |
| 4 | Training | 16134 patches | 2.69:1 | Patch class |
|  | Validation | 3228 patches | 3.90:1 | Patch class |
| 6 | Training | 16134 patches | 2.88:1 | Patch class |
|  | Validation | 3228 patches | 2.71:1 | Patch class |
| 9 | Training | 9684 patches | 5.3:1 | Patch class |
|  | Validation | 9678 patches | 1.77:1 | Patch class |


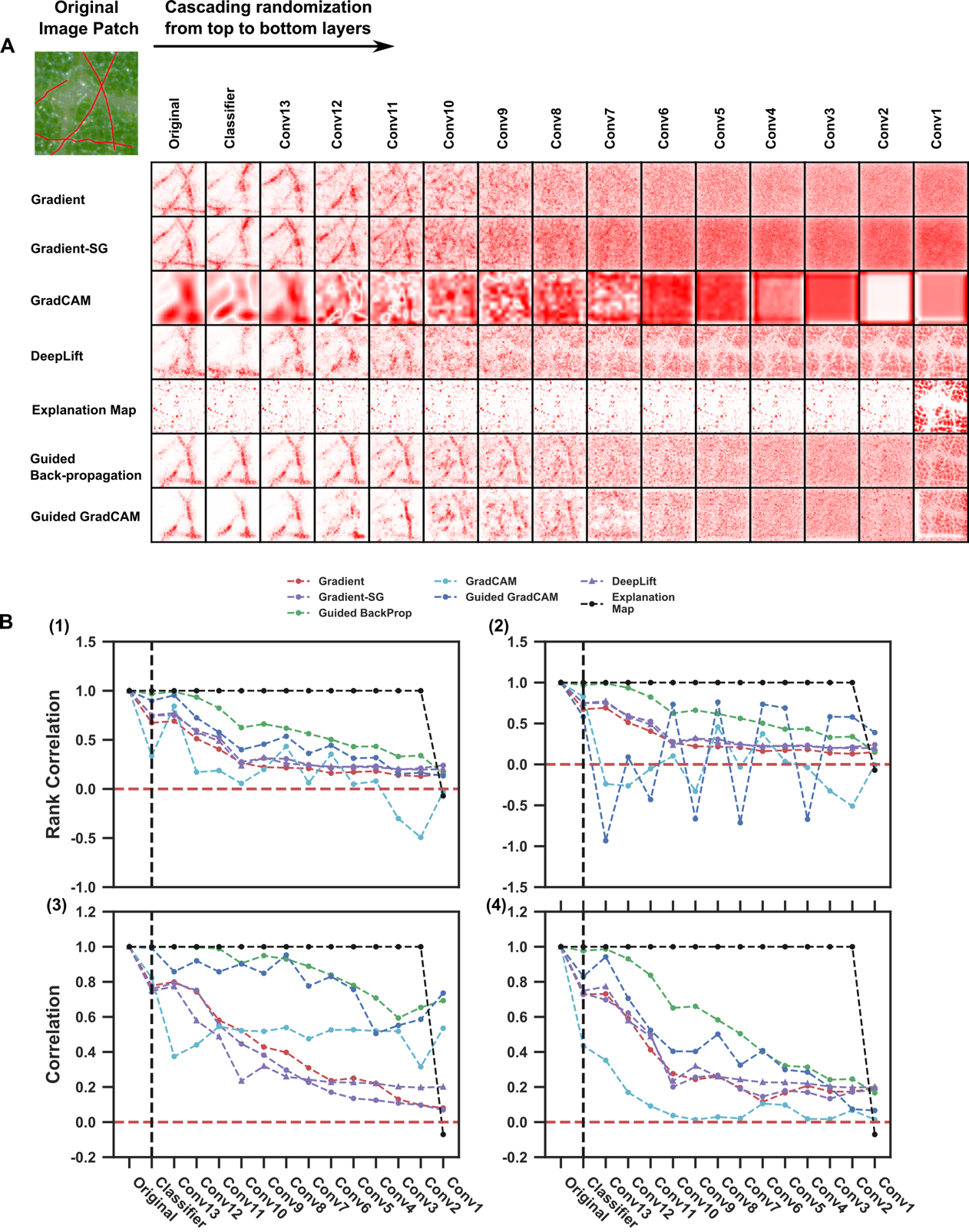


**Figure S2. Visualization of complete saliency maps and similarity metrics of sanity check with cascading layer randomization of VGG16. A: Saliency maps calculated for an input image using several saliency methods. B: Plots of various similarity metrics - (1) and (2) are Spearman Rank correlation with/without absolute values, (3) SSIM, (4) HOG.**


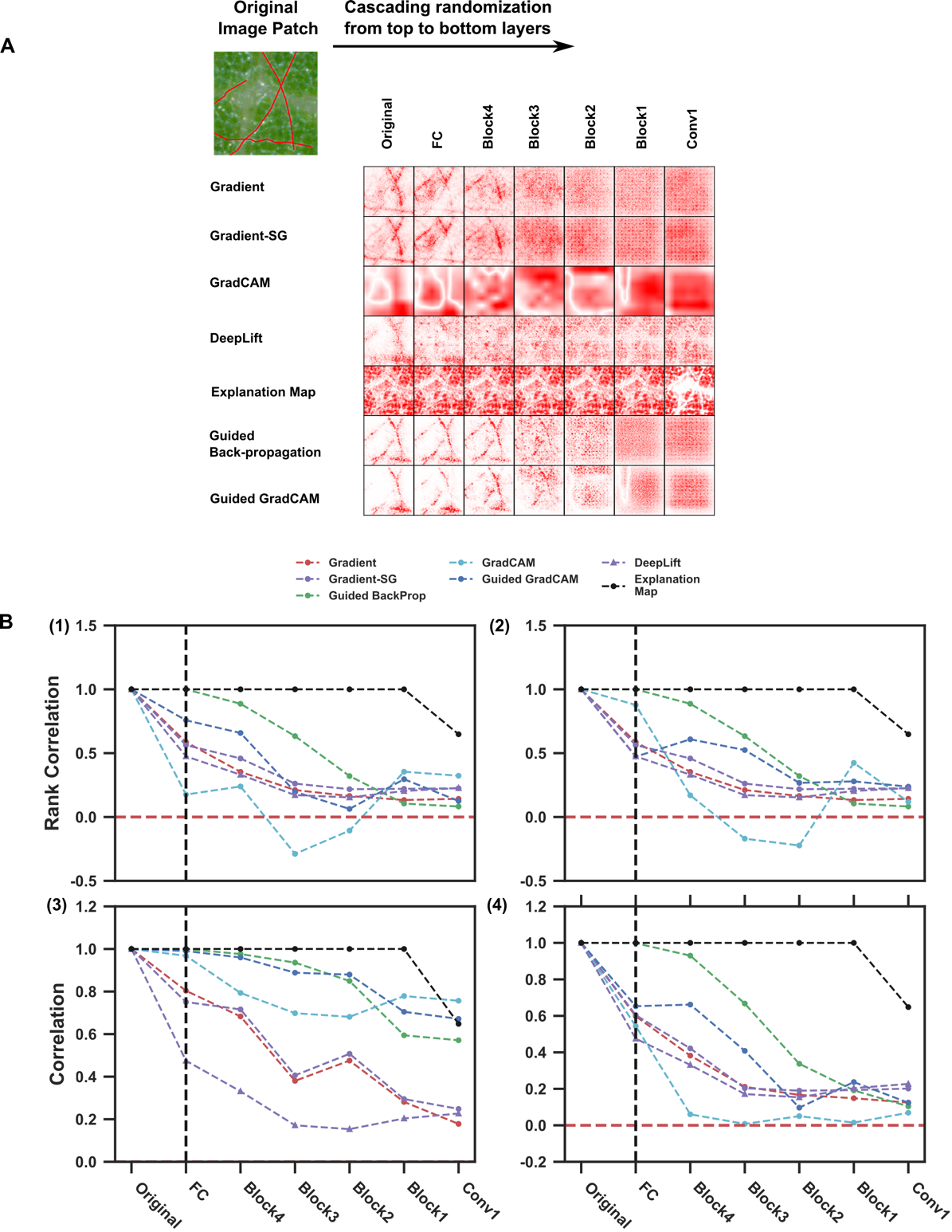


**Figure S3. Visualization of complete saliency maps and similarity metrics of sanity check with cascading layer randomization of ResNet50. A: Saliency maps calculated for an input image using several saliency methods. B: Plots of various similarity metrics - (1) and (2) are Spearman Rank correlation with/without absolute values, (3) SSIM, (4) HOG.**


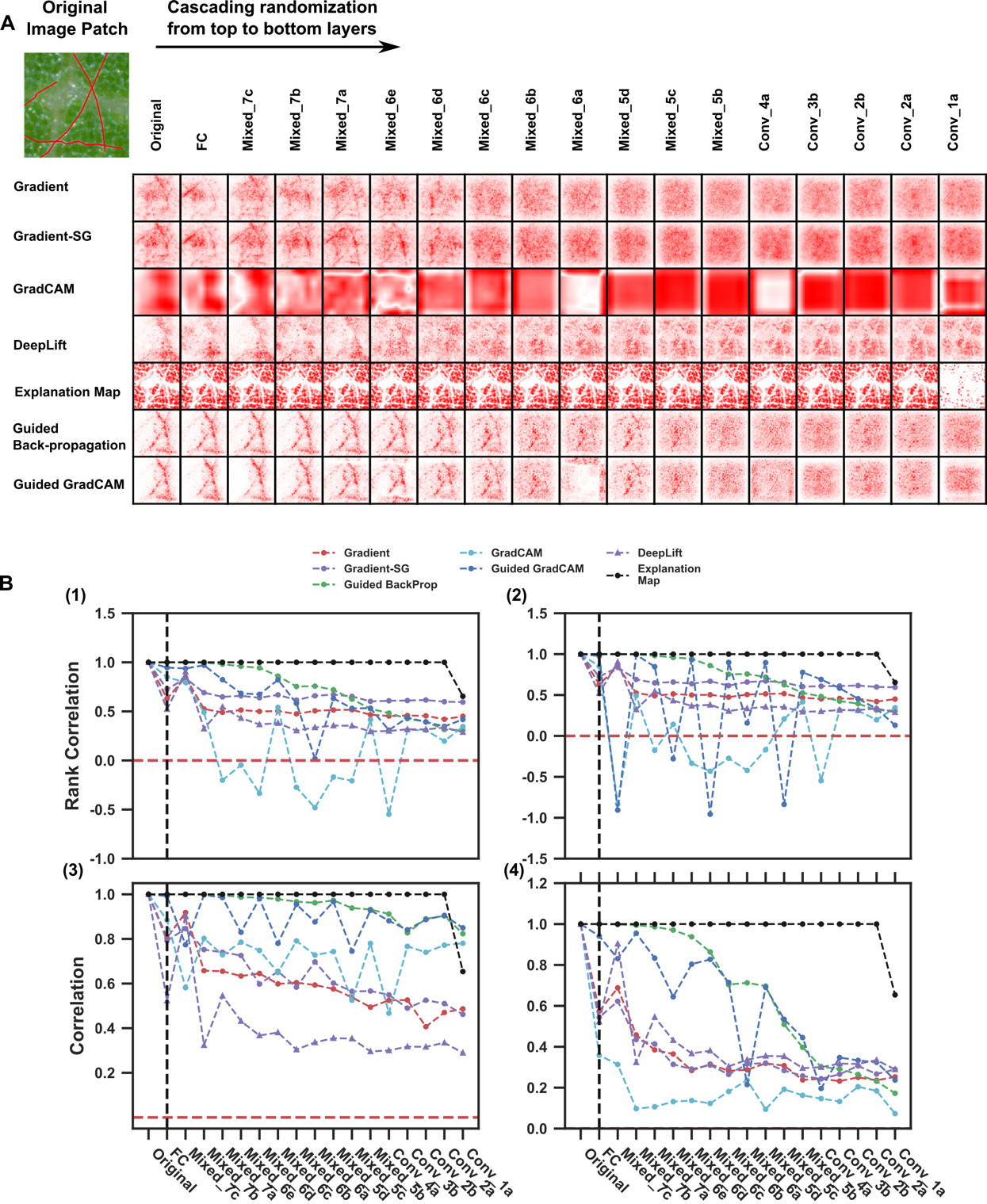


**Figure S4. Visualization of complete saliency maps and similarity metrics of sanity check with cascading layer randomization of Inception V3. A: Saliency maps calculated for an input image using several saliency methods. B: Plots of various similarity metrics - (1) and (2) are Spearman Rank correlation with/without absolute values, (3) SSIM, (4) HOG.**

Table S2. Classification accuracy and F1 score of the three CNN models on the validation and testing sets.

| Model | Classification accuracy | | F1 score | |
| --- | --- | --- | --- | --- |
|  | Validation | Testing | Validation | Testing |
| VGG16 | 95.53% | 93.33% | 0.9426 | 0.9048 |
| InceptionV3 | 95.07% | 92.89% | 0.9361 | 0.8989 |
| ResNet50 | 95.12% | 93.22% | 0.9373 | 0.9021 |

Table S3. Classification accuracy and F1 score of cross validation on the validation dataset using VGG16.

| Cross validation on i days after inoculation (DAI) | Classification accuracy | F1 score |
| --- | --- | --- |
| 1 | 99.35% | 0.6363 |
| 4 | 95.35% | 0.9287 |
| 6 | 96.28% | 0.9529 |
| 9 | 92.52% | 0.9183 |


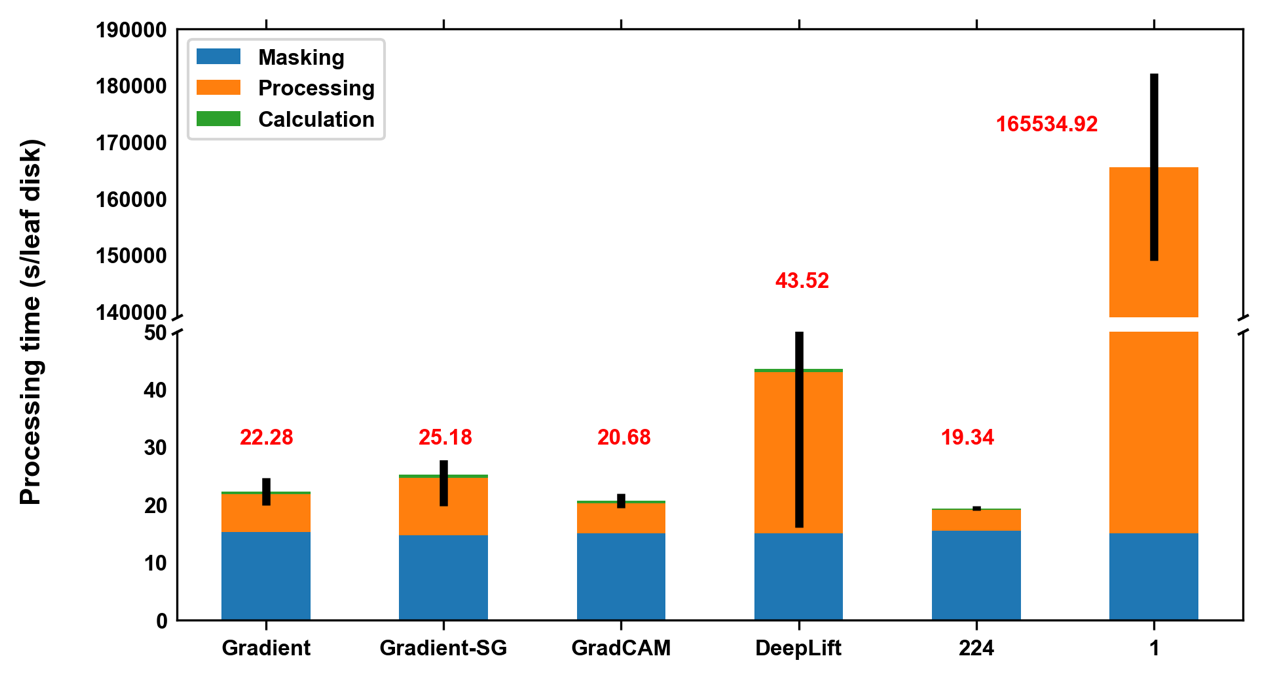


**Figure S5. Processing time for the saliency-based pipeline and the patch-based pipeline with different sliding window sizes. Gradient, Gradient-SG, GradCAM, and DeepLift were four specific saliency methods for the saliency-based pipeline. Numbers on x-axis indicated the sliding window sizes used by the patch-based pipeline. Mean values of the total processing time were marked in red color for all methods.**

Table S4. Average IoU and Dice score of DeepLab V3 for the infected class on the segmentation validation dataset in 10 repeated tests.

| Fold ID | (%) Intersection of Union (IOU) | (%) Dice |
| --- | --- | --- |
| 1 | 56.39 | 72.11 |
| 2 | 57.00 | 72.61 |
| 3 | 53.61 | 69.80 |
| 4 | 55.18 | 71.11 |
| 5 | 51.63 | 68.10 |
| 6 | 56.24 | 71.99 |
| 7 | 59.04 | 74.24 |
| 8 | 54.56 | 70.60 |
| 9 | 61.40 | 76.09 |
| 10 | 57.62 | 73.11 |
| Average ± Standard deviation) | 56.27±2.77 | 72.00±2.26 |

**Table S5. One-way ANOVA table for testing the PM quantification accuracy of different methods at the image patch level via 10 repeated tests.**

|  | Degree of freedom | Sum of squares | Mean of sum of squares | F value | Pr(>F) |
| --- | --- | --- | --- | --- | --- |
| Method | 2 | 0.00225 | 0.001126 | 0.218 | 0.805 |
| Residuals | 27 | 0.13940 | 0.005163 |  |  |

Table S6. Differences in infection rate calculated by each quantification pipeline between leaf samples in Figure 3A. The difference was calculated by comparing neighboring samples.

| Leaf sample | HT | | Classification-based | | Gradient-SG | | GradCAM | | DeepLab-V3 | |
| --- | --- | --- | --- | --- | --- | --- | --- | --- | --- | --- |
|  | Counts | RDT | IS (%) | RDT | IS (%) | RDT | IS (%) | RDT | IS (%) | RDT |
| Top | 290 | NA | 98.43 | NA | 30.32 | NA | 48.91 | NA | 9.2 | NA |
| Middle | 124 | 57% | 98.35 | 0.08% | 21.55 | 29% | 45.82 | 6% | 7.83 | 15% |
| Bottom | 68 | 45% | 88.73 | 10% | 15.92 | 26% | 29.56 | 35% | 4.07 | 48% |

Note: HT: Hyphal transect method for counting the number of hyphal using microscope, RDT: Relative difference

of infection severity to the previous leaf sample, IS: Infection severity.

**Table S7. One-way ANOVA table for the impact of quantification pipelines on Pearson’s correlation coefficients at the leaf sample level via 10 repeated tests.**

|  | Degree of freedom | Sum of squares | Mean of sum of squares | F value | Pr(>F) |
| --- | --- | --- | --- | --- | --- |
| Pipeline | 3 | 0.03286 | 0.010952 | 248.5 | <2e-16 *** |
| Residuals | 36 | 0.00159 | 0.000044 |  |  |

**Table S8. Tukey test for the impact of quantification pipelines on Pearson’s correlation coefficients at the leaf sample level via 10 repeated tests.**

| Quantification Pipeline | Difference | Lower confidence limit | Upper confidence limit | Adjusted p-value |
| --- | --- | --- | --- | --- |
| SAL-SG – SAL-GradCAM | 0.008138 | 0.0001422 | 0.01613 | 0.04477 |
| CLS – SAL-GradCAM | -0.06429 | -0.07228 | -0.05629 | 0 |
| SEG – SAL-GradCAM | -0.005179 | -0.01317 | 0.002816 | 0.3164 |
| CLS – SAL-SG | -0.07243 | -0.08042 | -0.06443 | 0 |
| SEG – SAL-SG | -0.01332 | -0.02131 | -0.005321 | 0.0004003 |
| SEG – CLS | 0.05911 | 0.051112 | 0.06710 | 0 |

Note: CLS: The classification-based pipeline, SAL-SG/GradCAM: The saliency-based pipeline with Gradient-SG/GradCAM, SEG: The segmentation-based pipeline.

**
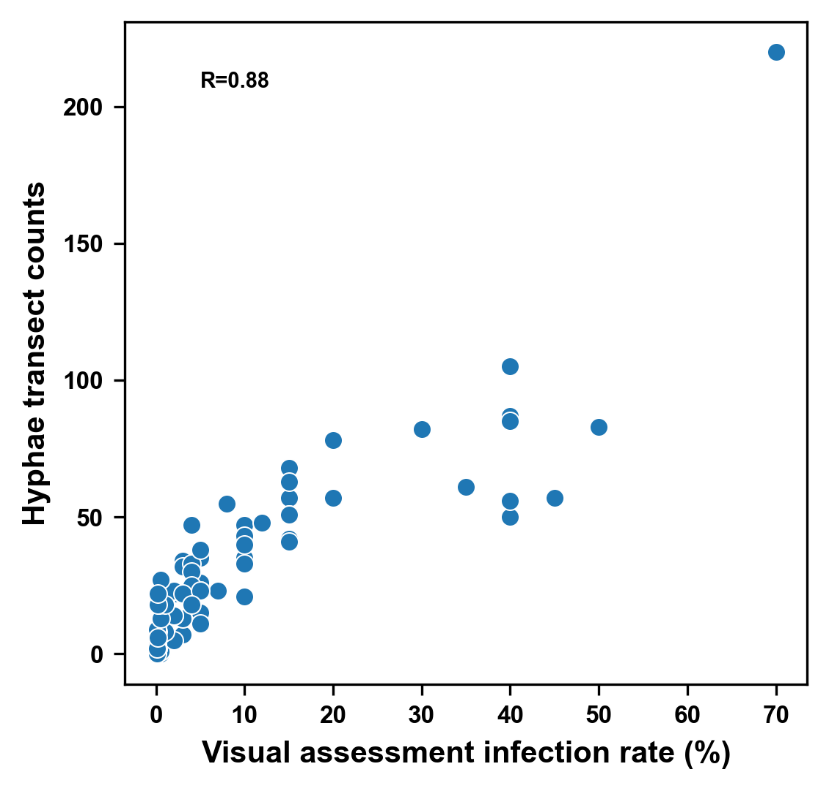
**

**Figure S6. Scatterplot of Pearson’s correlation coefficient between hyphae transect counts and manually visual assessment infection rate.**

**Table S9: A preliminary QTL mapping to test the explained phenotypic variance from different phenotypic traits generated by different quantification pipelines.**

| **Method** | **IS4** | | **IS6** | | **IS9** | | **Mean IS** | | **IG4-6** | | **IG6-9** | | **AUDPS** | |
| --- | --- | --- | --- | --- | --- | --- | --- | --- | --- | --- | --- | --- | --- | --- |
|  | **Chr 7** | **Chr 9** | **Chr 7** | **Chr 9** | **Chr 7** | **Chr 9** | **Chr 7** | **Chr 9** | **Chr 7** | **Chr 9** | **Chr 7** | **Chr 9** | **Chr 7** | **Chr 9** |
| **Classification-based** | **8.63** | **8.63** | **9.03** | **NA** | **7.98** | **NA** | **8.84** | **NA** | **7.43** | **NA** | **NA** | **NA** | **8.95** | **NA** |
| **GradCAM based saliency** | **8.89** | **9.06** | **9.66** | **8.7** | **9.14** | **NA** | **9.69** | **7.93** | **8.81** | **NA** | **NA** | **NA** | **9.71** | **8.32** |
| **Gradient-SG based saliency** | **8.73** | **8.59** | **9.35** | **8.63** | **8.73** | **NA** | **9.32** | **7.95** | **9.24** | **NA** | **NA** | **NA** | **9.35** | **8.26** |

Note: IS4/6/9: Infection severity on single 4/6/9 DAI, Mean IS: Mean infection severity from 4 to 9 DAI, IG4-6(IG6-9): Infection severity rate difference between 4(6) and 6(9) DAI, AUDPS: Area under disease progress stairs.

**Table S10: LOD threshold and scores for chromosomes (Chr) 7 and 9 using infection severity on 4 DAI (IS4), the most-effective trait, calculated by models trained with training datasets generated by different partitioning seeds.**

| Method | Partitioning Seed | LOD Threshold | LOD 7 | LOD 9 |
| --- | --- | --- | --- | --- |
| Patch-based | 1 | 4.23 | 4.48 | 5.07 |
|  | 2 | 4.17 | 4.69 | 4.95 |
|  | 3 | 4.2 | 4.44 | 5.18 |
|  | 4 | 4.09 | 4.3 | 5.08 |
|  | 5 | 4.27 | 4.69 | 4.87 |
|  | 6 | 4.2 | 4.69 | 5.02 |
|  | 7 | 4.2 | 4.59 | 5.07 |
|  | 8 | 4.15 | 4.49 | 4.81 |
| GradCAM | 1 | 4.21 | 4.38 | 5.38 |
|  | 2 | 4.25 | 4.71 | 5.13 |
|  | 3 | 4.12 | 4.45 | 5.49 |
|  | 4 | 4.13 | 4.33 | 5.38 |
|  | 5 | 4.22 | 4.82 | 5.24 |
|  | 6 | 4.16 | 4.81 | 5.4 |
|  | 7 | 4.22 | 4.52 | 5.43 |
|  | 8 | 4.15 | 4.59 | 5.35 |
| Gradient-SG | 1 | 4.19 | 4.34 | 5.12 |
|  | 2 | 4.12 | 4.68 | 4.82 |
|  | 3 | 4.18 | 4.48 | 5.03 |
|  | 4 | 4.1 | 4.33 | 5.01 |
|  | 5 | 4.25 | 4.74 | 4.89 |
|  | 6 | 4.21 | 4.7 | 4.97 |
|  | 7 | 4.13 | 4.45 | 5.14 |
|  | 8 | 4.2 | 4.55 | 4.82 |

**Table S11: LOD threshold and scores for chromosomes (Chr) 7 and 9 using mean infection severity (mean IS), a accuracy-sensitive trait, calculated by models trained with training datasets generated by different partitioning seeds.**

| Method | Partitioning Seed | LOD Threshold | LOD 7 | LOD 9 |
| --- | --- | --- | --- | --- |
| Patch-based | 1 | 4.22 | 4.77 | ***3.96*** |
|  | 2 | 4.21 | 4.9 | ***3.72*** |
|  | 3 | 4.16 | 4.8 | ***3.9*** |
|  | 4 | 4.16 | 4.74 | ***3.93*** |
|  | 5 | 4.14 | 4.96 | ***3.71*** |
|  | 6 | 4.29 | 4.86 | ***3.86*** |
|  | 7 | 4.09 | 4.91 | ***3.91*** |
|  | 8 | 4.23 | 4.8 | ***3.79*** |
| GradCAM | 1 | 4.2 | 5.13 | 4.5 |
|  | 2 | 4.2 | 5.25 | ***4.1*** |
|  | 3 | 4.22 | 5.18 | 4.53 |
|  | 4 | 4.06 | 5.07 | 4.52 |
|  | 5 | 4.16 | 5.36 | 4.33 |
|  | 6 | 4.23 | 5.26 | 4.55 |
|  | 7 | 4.21 | 5.34 | 4.38 |
|  | 8 | 4.24 | 5.25 | 4.42 |
| Gradient-SG | 1 | 4.15 | 5.17 | 4.39 |
|  | 2 | 4.3 | 5.2 | ***4.23*** |
|  | 3 | 4.11 | 5.01 | 4.56 |
|  | 4 | 4.16 | 5.01 | 4.47 |
|  | 5 | 4.11 | 5.12 | 4.32 |
|  | 6 | 4.13 | 5.08 | 4.43 |
|  | 7 | 4.16 | 5.1 | 4.41 |
|  | 8 | 4.16 | 5.08 | 4.21 |

**Table S12. One-way ANOVA test for the impact of the partition seed with the most effective phenotypic trait (IS4) on the phenotypic explanation.**

|  | Degree of freedom | Sum of squares | Mean of sum of squares | F value | Pr (>F) |
| --- | --- | --- | --- | --- | --- |
| Seed | 7 | 0.9781 | 0.1397 | 0.822 | 0.583 |
| Residuals | 16 | 2.7193 | 0.1699 |  |  |

**Table S13. One-way ANOVA test for the impact of the partition seed with the accuracy sensitive phenotypic trait (mean IS) on the phenotypic explanation.**

|  | Degree of freedom | Sum of squares | Mean of sum of squares | F value | Pr (>F) |
| --- | --- | --- | --- | --- | --- |
| Seed | 7 | 0.389 | 0.0556 | 0.071 | 0.999 |
| Residuals | 16 | 12.585 | 0.7866 |  |  |
